# UAV inspection path optimization in offshore wind farms using the OPTION-A*-DQN algorithm

**DOI:** 10.1371/journal.pone.0336935

**Published:** 2025-11-24

**Authors:** Meiqing Xu, Chao Deng, Xiangyu Hu, Yuxin Lu, Wenyan Xue, Bin Zhu

**Affiliations:** 1 School of Computer Science and Engineering, Guangdong Ocean University, Guangdong, China; 2 School of Mechanical and Energy Engineering, Guangdong Ocean University, Guangdong, China; 3 Three Gorges New Energy Yangjiang Power Generation Co., Ltd., Yangjiang, Guangdong, China; Beijing Institute of Technology, CHINA

## Abstract

In response to the inefficiencies in offshore wind farm inspections caused by path redundancy and mission omissions, this study proposes a novel path planning method for Unmanned Aerial Vehicle (UAV) inspections, integrating multi-constraint optimization and intelligent scheduling. First, a four-dimensional constraint model is established, encompassing wind speed, charging, minimum UAV fleet size, and dynamic obstacle avoidance. Second, the OPTION-A*-DQN hybrid algorithm is developed by synergizing A* heuristic search with deep reinforcement learning (DRL) to balance global navigation and local optimization. An improved K-Means algorithm further enables efficient topological partitioning for multi-UAV collaboration. Comparative evaluations against original OPTION-DQN and conventional heuristic methods (Dijkstra and Simulated Annealing) demonstrate that the proposed method achieves three key improvements: (1) a 10% higher task completion rate, (2) a 14.9% reduction in path distance, and (3) a 20% faster simulation time. This work significantly advances intelligent path planning for offshore wind farm inspections.

## Introduction

With the continuous expansion of offshore wind farm scales, traditional manual inspection methods have become increasingly inadequate due to their high cost, low efficiency, and safety risks, making them incapable of meeting the demand for high-frequency intelligent maintenance [1]. Unmanned Aerial Vehicles (UAVs) have become ideal inspection tools due to their strong mobility and sensing capabilities; however, their task efficiency and operational stability largely depend on the intelligence and effectiveness of the path planning system [2]. In complex maritime environments, challenges such as wind disturbances, energy constraints, and dynamic obstacles significantly increase the difficulty of path optimization [3]. Additional concerns, such as task allocation, obstacle avoidance robustness, and path redundancy, must be addressed in multi-UAV collaborative tasks, forming a typical multi-objective, multi-constraint problem [4]. Therefore, developing an adaptive and efficient UAV path planning system is critical for ensuring high-quality inspection.

Numerous studies have explored various path planning strategies to tackle these challenges. Among classical graph-based algorithms, Dijkstra’s algorithm offers global optimality [5] and performs well in static environments with complete information; however, it suffers from high computational complexity and limited responsiveness to dynamic changes. The A* algorithm introduces heuristic functions to balance search efficiency and path quality [6], and is widely used in intelligent navigation systems. Simulated Annealing (SA) [7] and Genetic Algorithms (GA) [8] possess strong global search capabilities, enabling them to escape local optima; however, they are susceptible to environmental perturbations in dynamic scenarios. Meta-heuristic approaches such as Ant Colony Optimization (ACO) [9], Particle Swarm Optimization (PSO) [10], and Artificial Potential Fields (APF) [11] have also been employed in path planning, yet commonly suffer from issues like unsmooth trajectories, slow convergence, or entrapment in local optima. Some studies have attempted to incorporate environmental awareness to enhance real-world applicability. For instance, an Improved Genetic Algorithm (IGA) proposed in [12] optimized inspection time and battery scheduling but lacked support for dynamic obstacle avoidance or swarm size management. The study in [13] considered wind effects and aimed to minimize the number of UAVs but failed to incorporate power management strategies. The Sea-Wind-Aware Improved A*-Guided Genetic Algorithm (SWA-IAGA) in [14] integrated obstacle avoidance but did not include a recharging mechanism, thereby limiting its applicability for long-duration missions. Neural network-based methods have also been explored. The study in [15] compared Transformer neural network models and simulated annealing algorithms for solving the Traveling Salesman Problem (TSP) under dynamic wind conditions but did not address UAV energy management. The study in [16] proposed a Graph Neural Network (GNN)-enhanced Multi-Agent Reinforcement Learning (MARL) framework to accelerate UAV swarm path planning; however, it did not consider energy constraints or real-world environmental factors. These methods typically address individual constraints in isolation and do not construct a cohesive optimization mechanism that considers wind speed, battery management, UAV fleet size, and dynamic avoidance simultaneously. Consequently, they are prone to path failures and task interruptions in real operations, especially under compounded constraints. For instance, obstacle avoidance may lead to longer paths and increased energy consumption, which in turn limits avoidance options, forming a vicious cycle known as cascading failures [17].

To improve adaptability to environmental uncertainties, Deep Reinforcement Learning (DRL) has gained attention due to its interactive and online learning capabilities [18,19]. The Deep Q-Network (DQN) [20], which combines neural networks with Q-value learning, has been widely applied to tasks such as path optimization [21,22], real-time obstacle avoidance [23], and energy scheduling [24]. However, basic DQN suffers from low exploration efficiency, unstable training, and fluctuating performance, particularly in large-scale collaborative missions, leading to redundant paths, slow convergence, and reduced task success rates. Consequently, enhanced architectures have been proposed, such as Double DQN (DDQN) [25], Prioritized Experience Replay DQN (PER-DQN) [26], and LSTM-integrated DQN (DQN-LSTM) [27], which improve training stability and temporal modeling. Multi-objective reward functions [28] have also been introduced to increase the model’s sensitivity to multiple constraints. However, these solutions have not overcome the core bottlenecks of slow policy convergence and unstructured task space modeling.

Hierarchical Reinforcement Learning (HRL) presents a promising approach to restructuring planning strategies. The OPTION mechanism decomposes complex tasks into multiple sub-policies, enabling improved learning efficiency and clearer policy hierarchies. OPTION-based frameworks have been successfully applied in robot path guidance [29], traffic signal control [30], and intelligent scheduling systems [31], especially in tasks with well-defined sub-goals. The key advantage lies in decoupling high-level task scheduling and low-level action learning, which enhances adaptability in dynamic scenarios. However, applying the OPTION framework to UAV path planning introduces new challenges. Without practical prior guidance, sub-policy transitions may become sluggish or redundant. Moreover, poorly structured sub-policy definitions can lead to training instability and performance degradation.

To address these limitations, this paper presents a systematic solution. First, a multi-constraint coupling optimization model is proposed, incorporating a four-dimensional constraint model that encompasses wind speed, recharging logistics, the minimum UAV fleet size, and real-time dynamic obstacle avoidance. Second, an intelligent UAV system for offshore infrastructure inspection with cooperative multi-agent planning is developed. At the algorithmic level, a novel OPTION-A*-DQN hybrid architecture is designed. The high-level OPTION module decomposes multi-constraint tasks, the mid-level A* component generates globally feasible trajectories under wind and power constraints, and the low-level DQN enables real-time decisions for dynamic obstacle avoidance. This integrated approach provides a theoretically rigorous and practically deployable solution for UAV inspections in complex environments.

The main contributions of this work are as follows:

1. By accounting for challenging maritime conditions and UAV operational constraints, this study established a four-dimensional constraint model.
2. This study developed a clustering-based inspection partitioning strategy using improved K-Means to optimize both UAV paths and overall task completion.
3. Simulation results confirm that OPTION-A*-DQN outperforms both OPTION-DQN and traditional heuristic methods (Dijkstra and SA) in terms of task completion, inspection efficiency, and stability.

## Environmental modeling of offshore wind farms

**Fig 1:**
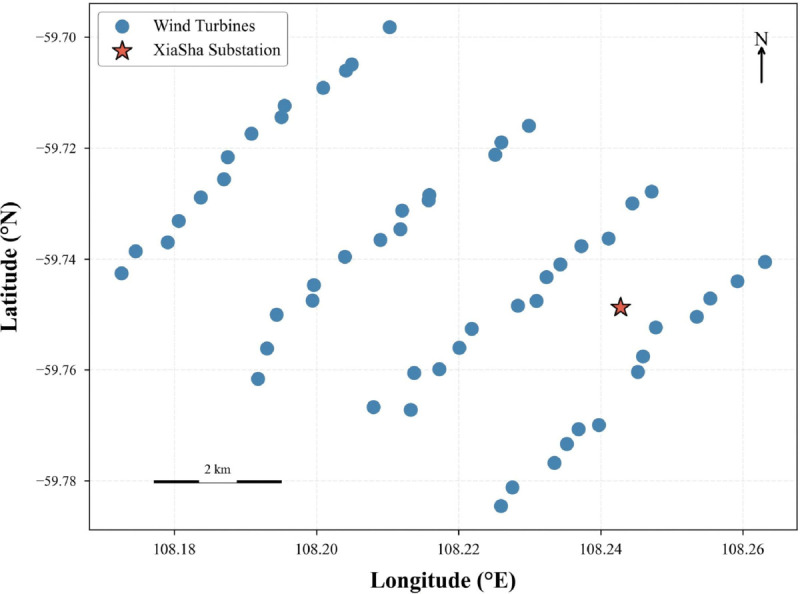
Turbine distribution map of Yangjiang Phase I wind farm.

This study chooses the Phase I area of the Three Gorges Yangjiang Shapa Offshore Wind Farm as the experimental setting. This location exemplifies modern, medium-to-large-scale offshore wind projects and poses a typical challenge for automated inspection. It includes 55 wind turbines and a central offshore substation that acts as the hub for UAV operations. The spatial arrangement of these assets, along with the complex maritime environment (such as dynamic wind fields), provides an ideal testbed for developing and testing robust multi-UAV path planning algorithms. The model incorporates the geospatial coordinates of all turbines and the substation, UAV flight restrictions, and task rules to create a realistic simulation environment for evaluating algorithms. Fig 1 shows the layout of the wind farm. The coordinates presented are desensitized after preprocessing.

## Problem modeling

### Four-dimensional constraint model

#### Wind speed.

The total number of turbines in the offshore wind farm is *T*, the coordinates of the kth turbine are $g=\left[x_{k},y_{k}\right]$, and the wind direction in the polar coordinate system containing wind speed information is represented as:

$$\theta_{w}^{pol}=\arctan\frac{w^{y}}{w^{x}}$$ (1)

where $\theta_{w}^{pol}$ is the wind direction in the polar coordinate system, which is defined differently from the wind direction in meteorological measurements, $w=\left[w^{x},w^{y}\right]$ is the wind speed, $w^{x}$ and $w^{y}$ and are the projections of the wind speed on the x-axis and y-axis, respectively.

In meteorological measurements, wind direction is represented by $\theta_{w}^{met}$, and 0, $\frac{\pi }{2}$, *π*, and $\frac{3\pi }{2}$ represent north, east, south, and west winds, respectively. This convention means that the wind direction angle increases clockwise, with north as the reference point (0^°^). In contrast, the standard mathematical polar coordinate system defines the angle as increasing counterclockwise from the positive x-axis (east). Therefore, the relationship between $\theta_{w}^{pol}$ and $\theta_{w}^{met}$ is as follows:

$$\theta_{w}^{pol}=\frac{3\pi }{2}-\theta_{w}^{met}$$ (2)

When the UAV flies to the wind turbine for inspection, it may encounter two wind conditions, i.e., downwind and upwind, and the wind conditions need to be considered in the decision-making of the UAV. Therefore, the synthetic velocity $S_{U,k_{1},k_{2}}$ of UAV U when flying from turbine $k_{1}$ to turbine $k_{2}$ is defined as:

$$S_{U,k_{1},k_{2}}\;=\;[S_{U,k_{1},k_{2}}^{x},S_{U,k_{1},k_{2}}^{y}]$$ (3)

Define the UAV speed $V_{{U,k}_{1},k_{2}}$ when UAV U flies from turbine $k_{1}$ to turbine $k_{2}$ as:

$$V_{U,k_{1},k_{2}}\;=\;[V_{U,k_{1},k_{2}}^{x},V_{U,k_{1},k_{2}}^{y}]$$ (4)

where $S_{U,k_{1},k_{2}}^{x}$ and $V_{U,k_{1},k_{2}}^{x}$ are projections on the x-axis and $S_{U,k_{1},k_{2}}^{y}$ and $V_{U,k_{1},k_{2}}^{y}$ are projections on the y-axis.

The UAV speed is the initial speed of the UAV, and the synthetic speed is the speed affected by the wind speed. The relationship between UAV velocity, wind speed, and synthetic velocity is expressed as:

$$S_{U,k_{1},k_{2}}=V_{U,k_{1},k_{2}}+w(t);$$ (5)

where *w*(*t*) = *API*_*weather*_(*t*) represents the wind speed information at the current time *t* obtained through the real-time weather data API.

Use $||V_{U,k_{1},k_{2}}|{|}_{2}$ and $||S_{U,k_{1},k_{2}}|{|}_{2}$ to denote air speed and ground speed, respectively. The maximum speed limit for UAV U is $V_{U}^{max}$. Usually, $V_{U}^{max}$ refers to the maximum airspeed value. However, if the UAV flies at a ground speed that is too high, its structural capacity may be reduced, and it may not remain stable. Therefore, the ground speed is limited to $V_{U}^{max}$ for a downwind condition. When the UAV faces a headwind, the air speed is limited to $V_{U}^{max}$. The angle between $S_{{U,k}_{1},k_{2}}$ and $V_{{U,k}_{1},k_{2}}$ is denoted by $\theta_{U,k_{1},k_{2}}^{S,V}$. In addition, $\theta_{U,k_{1},k_{2}}^{S,w}$ is used to denote the angle between *w* and $S_{{U,k}_{1},k_{2}}$. The maximum wind resistance for UAV U is denoted by $V_{U}^{wind}$.

The flight time of UAV U from turbine $k_{1}$ to turbine $k_{2}$ can be calculated as:

$$t_{U,k_{1},k_{2}}=\frac{||g_{k_{1}}-g_{k_{2}}|{|}_{2}}{||S_{U,k_{1},k_{2}}|{|}_{2}}$$ (6)

where $g_{k_{1}}$is the coordinates of the $k_{1}$ turbine, $g_{k_{2}}$ is the coordinates of the $k_{2}$ turbine, and $||g_{k_{1}}-g_{k_{2}}|{|}_{2}$ represents the Euclidean norm of the $g_{k_{1}}-g_{k_{2}}$ vector.

The UAV U also has a maximum flight time, denoted $t_{U}^{max}$, which represents the upper limit of the total flight time during the inspection.

#### Charging.

The agent only gets the overall instant reward $r_{\xi }$ of the OPTION at the end moment ξ of each OPTION. $r_{\xi }$ is a function of the OPTION initial state $s_{\xi }$ and the OPTION action $OP_{\xi }$, i.e., $r_{\xi }=r_{\xi }(s_{\xi },OP_{\xi })$. The overall instant reward of an OPTION is set to be divided into the power reward $r_{\xi }^{e}$, the inspection reward r${\;}_{\xi }^{c}$, and the path reward $r_{\xi }^{l}$ of the three parts, among which the power reward is primarily used to penalize the UAV for insufficient power during the execution of the OPTION, i.e.

$$r_{\xi }^{e}=\left\{ \begin{array}{c} N_{e},e_{\xi }\leq B\\ 0,e_{\xi }>0 \end{array}\right.$$ (7)

where *N*_*e*_ is a negative constant, when the power is less than or equal to the minimum power B, the UAV is given a larger penalty value to force it to return, and the minimum power B is the power required to support the UAV to return to charge.

Considering long-term performance degradation, the actual available capacity of the battery is modeled as:

$$B_{effective}=B_{0}(1-B^{*}\cdot N_{cycle})$$ (8)

where *B*_0_ is the nominal capacity of the battery, B^*^ is the attenuation coefficient, and *N*_*cycle*_ is the number of full cycle charges completed by the battery.

The inspection reward is used to penalize the UAV for repeatedly selecting the turbine OPTION that has completed the inspection, i.e.

$$r_{\xi }^{c}=\left\{ \begin{array}{c} N_{c},cr_{\xi }^{OP_{\xi }}=1\\ 0,cr_{\xi }^{OP_{\xi }}<1 \end{array}\right.$$ (9)

where *N*_*c*_ is a negative constant and $cr_{\xi }^{OP_{\xi }}=1$ indicates the inspection of a turbine that has already been inspected.

The path reward $r_{\xi }^{l}$ is inversely proportional to the distance $l_{\xi }$ flown by the UAV within that OPTION and is used to guide the UAV to learn to fly the shortest possible path to inspect the turbine, which can be expressed as

$$r_{\xi }^{l}=\frac{N_{l}}{l_{\xi }}$$ (10)

where *N*_*l*_ is a negative constant. Ultimately, the immediate reward $r_{\xi }$ received by the UAV for going through an OPTION is the sum of the above three rewards, denoted as

$$r_{\xi }=r_{\xi }^{e}+r_{\xi }^{c}+r_{\xi }^{l}$$ (11)

**Fig 2:**
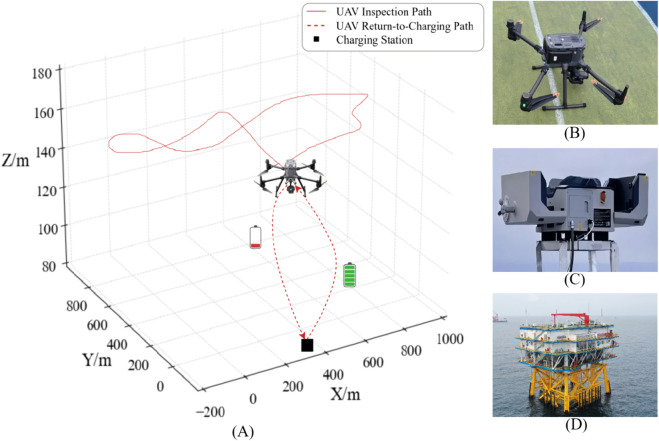
(A) The charging process of a UAV returning to the offshore substation for energy replenishment. (**B**) Industrial-grade UAV (DJI Matrice 350 RTK) used for autonomous inspection tasks. (**C**) Intelligent hangar (DJI Airport 2) serving as the automated UAV charging station. (**D**) An offshore substation equipped with the UAV charging infrastructure.

Fig 2 illustrates the integrated UAV inspection system deployed at the Three Gorges offshore substation. (A): The UAV automatically returns when battery drops below 20% and fast-charges to 90% within 45 minutes; (B): The UAV carries a high-resolution gimbal camera, infrared thermal imager, multispectral sensor, millimeter-wave radar, RTK-GPS, and a VPU for real-time crack detection; (C): The DJI Airport 2 hangar features IP55 protection, RTK positioning, automatic deployment/recovery, fast charging, edge computing, and wide-temperature operation; (D): The substation roof is reinforced with a carbon fiber platform, wind deflector, EMI shielding, southeast-aligned hangar layout, and fiber-optic data transmission.

#### Minimum UAV fleet size.

To ensure efficient UAV resource utilization, this constraint is implemented through a mathematical optimisation model to minimise the total number of UAVs required while satisfying the inspection needs of all turbines. Specifically, the total number of offshore wind turbines is *T*, and *M* is the set of available UAVs. The objective function for the minimum fleet size can be expressed as:

$$Minimize\;\sum_{U\in M}Y_{U}$$ (12)

where $Y_{U}$ is a binary variable indicating whether UAV U is being used. The constraint conditions include: each wind turbine must be inspected by at least one UAV, that is

$$\sum_{U\in M}X_{Uk}\geq 1,\forall k\in T$$ (13)

where $X_{Uk}$ is a binary decision variable indicating whether UAV U is assigned to inspect turbine *k*. At the same time, the inspection capability of each UAV is limited by its maximum endurance time and inspection task volume, that is

$$\sum_{k\in T}t_{k}X_{Uk}\leq T_{U},\forall U\in M$$ (14)

where $t_{k}$ denotes the time required for inspecting turbine *k*, and $T_{U}$ is the maximum endurance time of UAV U. At the same time, in order to extend the service life of the UAV fleet, the average loss coefficient of the fleet is introduced:

$$W_{avg}=\frac{1}{M}\sum_{U\in M}(W_{a}\cdot \frac{\sum_{k\in T}t_{k}X_{Uk}}{T_{U}}+W_{b}\cdot W_{count})$$ (15)

where *W*_*a*_ and *W*_*b*_ are weight coefficients, and *W*_*count*_ is the cumulative number of task cycles of UAV U.

With the above optimisation model, the paper achieves the minimisation of the number of UAVs to be used under the premise of meeting the demand for full-coverage inspection, thus reducing the inspection cost and improving the efficiency of resource utilisation.

#### Dynamic real-time obstacle avoidance.

**Fig 3:**
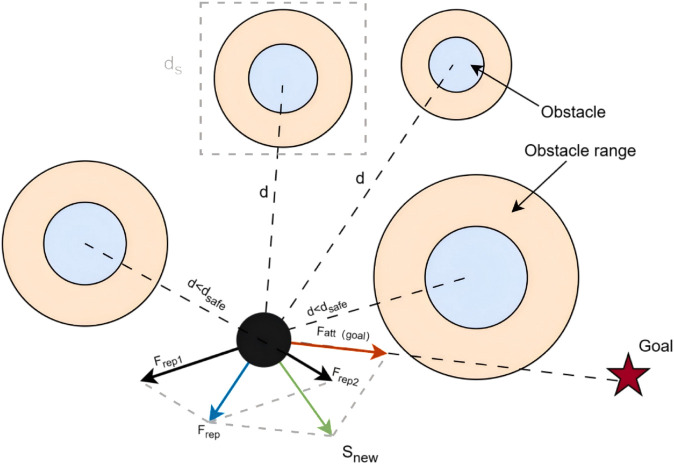
Illustration of dynamic real-time obstacle avoidance. The black dot represents the current UAV position, the red pentagram indicates the target location, the blue area denotes the core of a static obstacle, and the orange area represents the dynamic obstacle zone. The colored arrows show the planned avoidance directions in response to the presence of dynamic obstacles.

The system dynamically incorporates obstacle detection and avoidance mechanisms into the path planning process to enhance the flight safety and environmental adaptability of UAVs during inspection missions. Specifically, when a UAV detects the presence of nearby aerial objects (e.g., other UAVs, seabirds, etc.) within a predefined safety distance threshold $d_{safe}$, the avoidance strategy is triggered. The navigation system then adjusts the flight path to ensure uninterrupted task execution and maintain flight safety. Fig 3 illustrates the schematic diagram of the obstacle avoidance model based on the safety distance.

The sensor carried by the UAV can detect the distance to the dynamic obstacle as *d*. A safe distance threshold is defined as $d_{safe}$.

d=distance_sensor(ds) (16)

where *ds* denotes a dynamic obstacle.

The obstacle avoidance mechanism is triggered when the detected distance *d* is less than the safe distance threshold $d_{safe}$.

$$if\;d<d_{safe}\;then\;trigger\;avoidance$$ (17)

The obstacle avoidance function is defined as avoidance_function(s,d), where *s* is the current state of the UAV and *d* is the distance to a dynamic obstacle. The output of this function is an obstacle avoidance action *a*.

a=avoidance_function(s,d) (18)

Calculate the obstacle avoidance direction ${\vec{\nu }}_{avoid}$, which points away from the obstacle.

$${\vec{v}}_{avoid}=\frac{{\vec{p}}_{U}-{\vec{p}}_{obstacle}}{\left||{\vec{p}}_{U}-{\vec{p}}_{obstacle}|\right|}$$ (19)

where ${\vec{p}}_{U}$ is the position of the UAV and ${\vec{p}}_{obstacle}$ is the position of the obstacle.

Based on the obstacle avoidance direction, determine an obstacle avoidance action *a*, which can be a change in speed or direction.

$$a=normalize({\vec{v}}_{avoid}+{\vec{v}}_{current})$$ (20)

where ${\vec{\nu }}_{current}$ is the current velocity vector of the UAV, and the *normalize* function is used to normalise the vector to a suitable velocity magnitude.

An obstacle avoidance action is executed to adjust the path of the UAV.

$$s_{new}=update\_state(s,a)$$ (21)

where $s_{new}$ is the new state after performing the obstacle avoidance action.

### Cooperative multi-UAV mechanism

#### Intelligent inspection area partitioning strategy.

**Fig 4:**
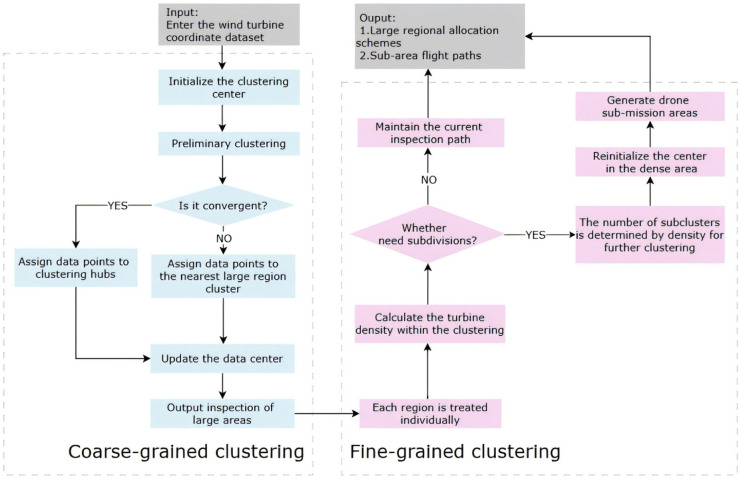
Flowchart of the intelligent inspection area partitioning algorithm.

This study employs the K-Means algorithm to partition the UAV inspection area. The OPTION-A*-DQN algorithm is then used to generate inspection paths for each subdivided region. This approach transforms the multi-UAV collaborative inspection problem into a set of single-UAV inspection tasks within individual subregions, effectively avoiding the path redundancy issues commonly encountered in traditional multi-UAV inspection methods, often resulting in low completion rates. Consequently, the proposed method significantly improves the overall inspection efficiency. Fig 4 illustrates the overall workflow of the intelligent inspection area partitioning framework proposed in this study.

Minimise the following objective function:

$$J=\sum_{i=1}^{\rho }\sum_{x\in \sigma_{i}}||x-\varphi_{i}|{|}^{2}$$ (22)

where *ρ* is the number of clusters, which corresponds to the number of divisions of the inspection turbine range, $\sigma_{i}$ is the ith cluster, which contains all the turbines belonging to the cluster, $\varphi_{i}$ is the centre of mass (centroid) of the *i*th cluster, which is usually the mean value of all the points within the cluster. *x* is the coordinate vector of a turbine within the cluster. The term $||x\;-\;\varphi_{i}|{|}^{2}$ is the square of the Euclidean distance from a turbine coordinate *x* to the centroid $\varphi_{i}$ of its cluster. The objective function *J* therefore represents the total within-cluster sum of squares, which the K-Means algorithm aims to minimize to form compact and well-separated clusters.

Secondly, the K-Means algorithm is improved using multiscale clustering considering the minimum cluster size. This is because multi-scale clustering allows for resource allocation at different levels, which can effectively reduce the number of UAVs required while ensuring mission completion.

Coarse-grained clustering is performed first, dividing neighbouring turbines into larger clusters, and then fine-grained clustering is performed within these clusters. This allows fewer UAVs to be used for inspections in larger clusters and allows UAVs to cover more turbines within a cluster.

Coarse-grained clustering: initial clustering using a large distance threshold

$$C_{j}=\{x|d(x,m_{j})<=T\_coarse\}$$ (23)

where $C_{j}$ is the jth cluster, $m_{j}$ is the centre of mass of the cluster $C_{j}$, and T_coarse is the threshold for coarse-grained clustering.

Fine-grained clustering: perform fine-grained clustering within each coarse-grained cluster

$$C_{ij}=\{x|d(x,m_{ij})<=T\_fine\}$$ (24)

where $C_{ij}$ is the ith fine-grained cluster within the jth coarse-grained cluster, $m_{ij}$ is the centre of mass of the cluster $C_{ij}$, and T_fine is the threshold for fine-grained clustering.

Multi-scale clustering helps to reduce the overlap of UAV inspection paths, thus reducing unnecessary flight time and energy consumption. The distribution of offshore turbines can be uneven, and multi-scale clustering can better accommodate this variation by ensuring that more resources are used in dense turbine areas and less in sparse areas.

#### Modeling of the inspection path optimization problem.

Following region partitioning and task assignment, UAVs are deployed from the intelligent hangar at the offshore substation to inspect designated wind turbines. The path optimization is modeled as a Family Traveling Salesman Problem (FTSP), where multiple UAVs start from a common origin, collaborate to access various target points, and ultimately return to the starting point. The goal is to minimize flight time and energy consumption while ensuring full coverage and improving inspection efficiency.

The system uses an asymmetric adjacency matrix to determine optimal flight sequences based on shortest-path criteria. Integrated real-time energy monitoring and a dynamic return mechanism guarantee operational continuity: when battery levels fall below a set threshold, UAVs autonomously return to the hangar for recharging, thus maintaining inspection reliability.

## OPTION-A*-DQN algorithm

This paper presents a multi-UAV collaborative path planning method for offshore wind farm inspection using the OPTION-A*-DQN algorithm. This hybrid architecture systematically combines hierarchical reinforcement learning with heuristic search. The framework includes three coordinated layers: a high-level OPTION module for task decomposition and temporal abstraction, a mid-level A* planner for generating globally feasible paths that account for wind and energy constraints, and a low-level DQN controller for real-time obstacle avoidance and trajectory fine-tuning. By leveraging the interpretability and optimality of A*, the adaptive learning of DQN, and the structured policy abstraction provided by the OPTION mechanism, the algorithm effectively addresses the challenges of high-dimensional action spaces, sparse rewards, and dynamic environmental uncertainties. This integration not only speeds up training convergence and improves sample efficiency but also enhances generalization across large-scale multi-UAV inspection scenarios, achieving a balanced trade-off among path optimality, task completion, and operational reliability.

### OPTION-DQN: A hierarchical reinforcement learning approach based on temporal abstraction

OPTION-DQN is a state-of-the-art reinforcement learning algorithm that extends the traditional deep Q-network (DQN) framework by introducing a temporal abstraction (TAB) mechanism. The algorithm combines the OPTION framework to solve the problems of long-term credit allocation sparsity and reward sparsity of traditional flat reinforcement learning methods in complex environments. The core idea of OPTION-DQN is to combine high-level actions (i.e., ‘OPTION’) with the underlying primitive actions to enable intelligence to make decisions at different time scales. OPTION is defined by the triad (I,π,β), where I denotes the initial set of states (the states in which an OPTION can be invoked), *π* is the internal policy (state-to-action mapping), and *β* is the termination condition (the probability that an OPTION will terminate in a particular state). By learning both OPTIONs and primitive actions simultaneously, intelligences can explore the environment more efficiently and optimise decisions over multiple time scales.

**Fig 5:**
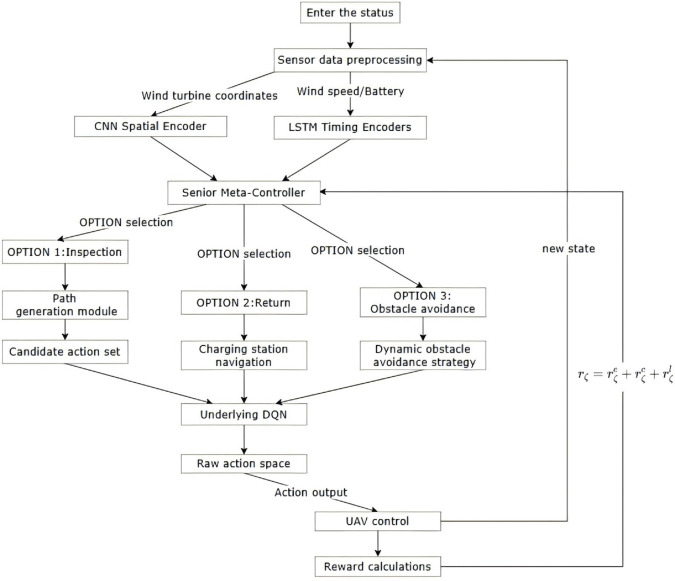
OPTION-DQN algorithm flowchart.

OPTION-DQN employs a dual Q-network architecture to learn the value functions of primitive actions and the OPTION, respectively. Specifically, the Q-primitive network estimates the value of primitive actions, while the Q-OPTION network evaluates the execution value of OPTIONs. During training, the intelligences ensure that the two levels of abstraction can be optimised synergistically by alternately exploring OPTIONs and optimising their internal policies. In addition, OPTION-DQN introduces an OPTION discovery mechanism to enhance the learning efficiency by identifying useful subgoals and their corresponding OPTIONs through intrinsic motivation or unsupervised learning. Meanwhile, the algorithm adopts an option-critic architecture to learn OPTION strategies and their termination conditions directly via gradient descent, enabling end-to-end training. Fig 5 shows the overall flow of the OPTION-DQN algorithm.

### A* Algorithm

In the OPTION-A*-DQN algorithm, the A* heuristic algorithm can, on the one hand, generate static a priori path information and Q-tables, which as a priori information reduces the initial exploration time and computational resources of the OPTION-A*-DQN algorithm, and improves the efficiency and stability at the early stage of training; on the other hand, it can reasonably adjust the weights of the a priori knowledge Q-value and estimated Q-value to generate the overall Q-value of the system. It achieves collaborative optimisation among agents, further optimises the action exploration strategy and multi-objective reward function, balances the exploration and exploitation relationship, and improves the autonomous decision-making ability of the UAV, to be more effectively applied to the complex environment of multiple UAVs [6].

### OPTION-A*-DQN

The OPTION-A*-DQN algorithm execution architecture demonstrates the reasoning process during the execution of the algorithm, where the UAV exits from the previous OPTION $OP_{\xi -1}$ during its interaction with the environment, obtaining the overall instantaneous reward $r_{\xi -1}$ for that OPTION as well as the state information for the next step as $s_{\xi }$, which $s_{\xi }$ consists of a vector of the percentage of the data that all the current wind turbines have already captured, $cr_{\xi }$, the UAV’s positional information $p_{\xi }$, as well as the UAV’s remaining electricity $e_{\xi }$. In turn, the current $s_{\xi }$ is input into the value function neural network *Q*_*ot*_ in the OPTION-A*-DQN algorithm, which consists of an input layer, a hidden layer, and an output layer, where the hidden layer consists of two fully-connected layers, and the first fully-connected network contains 1,024 neurons. Its activation function adopts the ELU (Exponential Linear Unit) function, which has the property of exponential decay in the negative domain. It can provide a smooth gradient and help accelerate the training process. The output of the first layer network can be expressed as

$$X_{1}=ELU(W_{1}^{T}s_{\xi }+b_{1})$$ (25)

where *W*_1_ is the weight parameter of the first layer neural network and *b*_1_ is its deviation parameter. The input of the second hidden layer is the output of the first hidden layer. The second hidden layer consists of 300 neurons, and its activation function is the same as that of the previous layer, which also uses the ELU function. The output of this layer can be expressed as

$$X_{2}=ELU(W_{2}^{T}X_{1}+b_{2})$$ (26)

where *W*_2_ and *b*_2_ are the second layer network’s weight and deviation parameters, respectively. The output layer accepts the output of the second layer network and utilizes the Softmax activation function to output the |O| dimensional vector *q* as

$$q=Softmax(W_{3}^{T}X_{2}+b_{3})$$ (27)

where *W*_3_ and *b*_3_ are the output layer’s weight parameter and deviation parameter, respectively. Softmax is a normalized exponential function that converts the neural network’s output into a probability distribution.

Finally, the output *Q*_*ot*_ of the value function neural network $𝐪=({𝐪}_{1},{𝐪}_{2},\cdots ,{𝐪}_{\left|o\right|})$ is the probability of selecting each OPTION, i.e., $\sum_{j=1}^{\left|O\right|}q_{j}=1$.

The optimal OPTION is found using the ε−greedy algorithm. *ε* is a smaller value from 0 to 1. Each time, a random selection is made with *ε* probability, and the selection is made using the greedy algorithm with 1−ε probability, i.e., the index of the largest value in *q* is chosen as the OPTION to be selected $OP_{\xi }$, and the greedy algorithm can be expressed as

$$OP_{\xi }=arg\max(q_{j})$$ (28)

Finally, the $OP_{\xi }$ corresponding strategy $\pi_{\xi }$ and termination condition $\beta_{\xi }$ are selected in the OPTION set *O*, and the corresponding action is output to continue the interaction with the environment.

The training algorithm for OPTION-A*-DQN is as follows:

In training the OPTION-A*-DQN algorithm, the UAV experience memory $𝒟=\{e_{k}|k\in D_{c}\}$ is set up, where *e*_*k*_ is the kth experience vector, i.e., $e_{k}=({\tilde{s}}_{k},{\tilde{o}}_{k},{\tilde{r}}_{k},{\tilde{s}}_{k}^{\prime })$, ${\tilde{s}}_{k}$ denotes the current state, ${\tilde{o}}_{k}$ denotes the OPTION action obtained according to the current OPTION-A*-DQN algorithm, ${\tilde{r}}_{k}$ is the overall immediate feedback received from $\left(s_{k}^{\prime },o_{k}^{\prime }\right)$, and ${\tilde{s}}_{k}^{\prime }$ and $o_{k}^{\prime }$ denote the next UAV transferring after the interaction with the environment a state and action, while *D*_*c*_ indicates the memory maximum storage. The use of empirical playback and random sampling to train the value function neural network *Q*_*ot*_ can break the correlation between the data and make the data satisfy the independent and identically distributed properties as much as possible, thus enhancing the stability of the training. The value function neural network *Q*_*ot*_ in the algorithm is also called the evaluation network. In addition, the target network $Q_{ot}^{Target}$ is set to be used to approximate the optimal evaluation network $Q_{ot}^{*}$, i.e., $Q_{ot}^{\text{Target}}\approx Q_{ot}^{*}$. The loss function of the evaluation network can be expressed as

$$Z(\theta )=E_{({\tilde{s}}_{k},{\tilde{o}}_{k},{\tilde{r}}_{k},{\tilde{s}}_{k}^{\prime })\sim D}[(\gamma argmaxQ_{ot}^{Target}({\tilde{s}}_{k}^{\prime })+{\tilde{r}}_{k}-Q_{ot}({\tilde{s}}_{k},{\tilde{o}}_{k};\theta )){]}^{2}$$ (29)

where *θ* represents all the parameters in the value function neural network *Q*_*ot*_ with the update rule of

$$\theta_{New}=\theta_{Old}-\alpha \nabla_{\theta }𝒵(\theta )$$ (30)

where *α* is the learning rate, $\theta_{New}$ and $\theta_{Old}$ denote the parameters after and before the update of the evaluation network, respectively, and the gradient of the loss function $\nabla_{\theta }𝒵(\theta )$ can be expressed as

$$\nabla_{\theta }𝒵(\theta )=E_{({\tilde{s}}_{k},{\tilde{o}}_{k},{\tilde{r}}_{k},{\tilde{s}}_{k}^{\prime })\sim D}[2(argmaxQ_{ot}^{Target}({\tilde{s}}_{k})+{\tilde{r}}_{k}-Q_{ot}({\tilde{s}}_{k},{\tilde{o}}_{k};\theta ))\;\times \nabla_{\theta }Q_{ot}({\tilde{s}}_{k},{\tilde{o}}_{k};\theta )]$$ (31)

In order to get the overall instant feedback, it is indicated that after interacting with the environment to take Soft-Update way to update the target network, i.e., after every certain period, the parameters of the original target network and the current estimation network are used to update the target network together, and its update rule is

$$\theta_{Target,New}=\beta \theta_{Target,Old}+(1-\beta )\theta$$ (32)

where *β* is the update rate, and β∈[0,1], $\theta_{Target,New}$ and $\theta_{Target,Old}$ denote the parameters of the target network $Q_{ot}^{Target}$ after updating and before updating, respectively, and adopting the Soft-Update approach to the target network can increase the robustness of neural network training.

The composition of the OPTION-A*-DQN algorithm is presented in Algorithm 1.

Algorithm 1 OPTION-A*-DQN for Multi-UAV Path Planning

**Input:** Wind turbine set $T=\{T_{1},...,T_{N}\}$; UAV status $s_{\xi }=(cr_{\xi },p_{\xi },e_{\xi })$;

A* heuristic function f(n)=g(n)+h(n);

Offshore wind farm boundaries and terrain map Ω;

UAV flight constraints: maximum range, flight speed, and energy capacity.

**Output:** Optimized inspection path *P* and High-Level OPTION policy $OP_{\xi }$.

**Initialize:** Global Q-network $Q_{\theta }$ and target network $\theta^{\text{Target}}$; Experience replay buffer *D* for OPTION policy *μ* (High-Level) and action policy *π* (Low-Level).

1: **for** each episode = 1 to *M* **do**

2:   Initialize the environment

3:   **while** inspection task is not completed **do**

4:     **High-Level Decision (OPTION Selection):**

5:     **if** remaining energy $e_{\xi }<20\%$ **then**

6:       Set $OP_{\xi }=\text{Return}$

7:     **else**

8:       **if** random sample <ε **then**

9:         Select random OPTION $OP_{\xi }$

10:       **else**

11:         $OP_{\xi }=\arg\max q(s_{0})$

12:       **end if**

13:     **end if**

14:     Mid-Level Guidance (A*-Based Planning):

15:     **if** $OP_{\xi }=$ Inspect **then**

16:       Compute path $P_{\text{A}}$ using A* from current location to

target

17:       Discretize $P_{\text{A}}$ to obtain action sequence *A*

18:     **else if** $OP_{\xi }=$ Avoid **then**

19:       Generate avoidance actions *A* using obstacle_avoid(*s*_0_)

20:     **end if**

21:     **Low-Level Control (DQN Execution):**

22:     Input state ${\tilde{s}}_{k}$ and proposed action *A* into $Q_{\theta }$

23:     Select optimal action ${\tilde{o}}_{k}$

24:     Execute ${\tilde{o}}_{k}$, observe reward ${\tilde{r}}_{k}$ and next state ${\tilde{s}}_{k}^{\prime }$

25:     **Network Update:**

26:     **if** |D|> batch_size **then**

27:       Sample mini-batch *C* from *D*

28:       **for** each $\left({\tilde{s}}_{k},{\tilde{o}}_{k},{\tilde{r}}_{k},{\tilde{s}}_{k}^{\prime }\right)$ in *C* **do**

29:         Compute loss:


$$𝒵(\theta )={𝔼}_{({\tilde{s}}_{k},{\tilde{o}}_{k},{\tilde{r}}_{k},{\tilde{s}}_{k}^{\prime })\sim 𝒟}[(\gamma argmaxQ_{ot}^{Target}({\tilde{s}}_{k}^{\prime })+{\tilde{r}}_{k}-Q_{ot}({\tilde{s}}_{k},{\tilde{o}}_{k};\theta )){]}^{2}$$

30:         Update gradient:


$$\nabla_{\theta }𝒵(\theta )={𝔼}_{({\tilde{s}}_{k},{\tilde{o}}_{k},{\tilde{r}}_{k},{\tilde{s}}_{k}^{\prime })\sim 𝒟}[2(argmaxQ_{ot}^{Target}({\tilde{s}}_{k}^{\prime })+{\tilde{r}}_{k}-Q_{ot}({\tilde{s}}_{k},{\tilde{o}}_{k};\theta ))\nabla_{\theta }Q_{ot}({\tilde{s}}_{k},{\tilde{o}}_{k};\theta )]$$

31:         Soft update target network: $\theta_{\text{Target,New}}\leftarrow \beta \theta_{\text{Target,New}}+$

(1−β)θ

32:     **end if**

33:   **end while**

34: **end for**

### Algorithm-hardware integration analysis

The hardware setup of the UAV, especially sensor accuracy and communication latency, is crucial in determining the performance of the proposed OPTION-A*-DQN algorithm. The high-precision RTK-GPS (2 cm accuracy) provides accurate positional feedback, which is vital for reliable state representation in the reinforcement learning framework. Low-latency VPU processing (15 ms) enables real-time obstacle avoidance and rapid policy updates, thereby improving the algorithm’s responsiveness in dynamic environments. Additionally, the multisensor suite (61-megapixel camera, infrared imager, and millimeter-wave radar) offers comprehensive environmental perception, supporting robust feature extraction and state transitions within the DQN network. Any decline in sensor accuracy or increase in communication delay could negatively impact state estimation, reward calculation, and action selection, thereby affecting path optimality, training convergence, and overall task success.

## Simulation results

### Experimental setup and environment configuration

All simulation experiments were conducted on the same computing platform to ensure the comparability of results. The hardware configuration of the experimental platform includes an Intel^®^ Core i5-13600K CPU @ 3.50 GHz, an NVIDIA GeForce RTX 2080 Ti GPU, and 32 GB of RAM, running on the Windows 11 operating system. The algorithms were implemented in Python 3.9, with the training process carried out using the PyTorch framework for deep reinforcement learning modeling and path simulation. Simulation visualization was performed using the Matplotlib and OpenCV libraries.

This study aims to evaluate the path planning performance of the improved OPTION-DQN algorithm enhanced with heuristic search mechanisms in complex offshore wind farm environments. The simulation experiments are conducted in two phases: the first phase involves simulation testing, where a simplified wind farm model is constructed based on 30 randomly generated wind turbine coordinates; the second phase comprises engineering testing, utilizing the actual coordinates of 55 wind turbines from the Yangjiang Phase I offshore wind project for full-coverage path optimization. Evaluation metrics include inspection coverage rate, path length, task duration, return-to-charge frequency, and training stability.

All path planning parameters are standardized to ensure the scientific rigor and reproducibility of the experiments. Table 1 provides a detailed overview of the algorithm parameters, UAV dynamics parameters, path planning settings, and experimental configurations used in this study. All improved algorithms are executed under identical parameter settings to eliminate performance variability caused by parameter bias.

**Table 1:** Simulation parameters for UAV path planning.

Parameter Name	Parameter Symbol	Parameter Value
Iterations	*T*	1200
Learning Rate	*α*	0.0001
Discount Factor	*γ*	0.99
Exploration Rate	*ε*	1.0 → 0.01
Replay Buffer	*D*	10000
Batch Size	*B*	32
Max Speed	$v_{\max}(m/s)$	5.0
Max Acceleration	$a_{\max}({m/s}^{2})$	0.5
Initial Battery	*E*_0_	100.0
Battery Capacity	*E*_*t*_(min)	30-40
Time Step	Δt(s)	0.1
A* Heuristic Weight	*w*	1.2
Max Return Distance	$d_{\max}(km)$	12
Random Seed	seed	42

### Evaluation of the improved algorithm design

To enhance the efficiency of multi-UAV collaborative operations, this experiment employs an improved K-Means clustering algorithm to partition the 55 wind turbines of the Yangjiang Phase I offshore wind farm into three subregions with balanced topology and reasonable task loads. Each subregion is independently assigned to a single UAV for inspection. When the UAV’s battery level falls below a predefined threshold, it automatically returns to the offshore substation for recharging before resuming its task, ensuring operational continuity and effective energy management. This regional division strategy is implemented during the modeling phase and remains fixed throughout the experiments. Each UAV is restricted to its designated subregion, which effectively prevents traditional issues in multi-agent systems such as path intersection, task conflict, and redundant inspection, thus providing a stable foundation for evaluating the performance of the path optimization algorithms.

Based on this regional division, three heuristic algorithms (Dijkstra, Simulated Annealing, and A*) are integrated with the OPTION-DQN framework to construct three improved models: OPTION-Dijkstra-DQN, OPTION-SA-DQN, and OPTION-A*-DQN.

**Fig 6:**
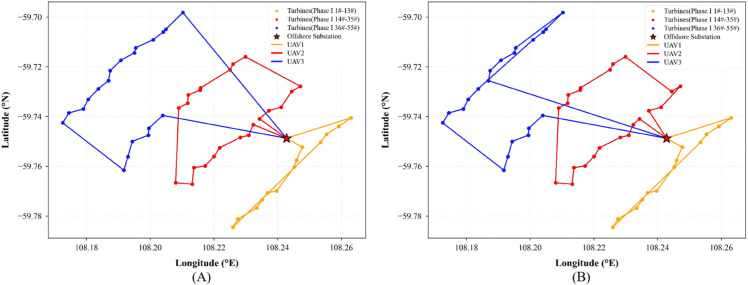
(A) Initial path planning result using the OPTION-Dijkstra-DQN algorithm. (**B**) Optimized path generated by the improved OPTION-Dijkstra-DQN algorithm.

**Fig 7:**
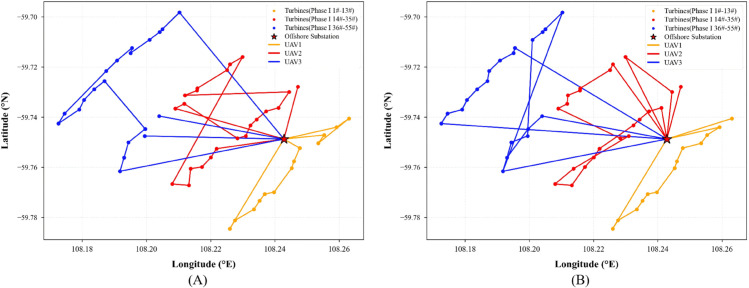
(A) Initial path planning result using the OPTION-SA-DQN algorithm. (**B**) Optimized path generated by the improved OPTION-SA-DQN algorithm.

**Fig 8:**
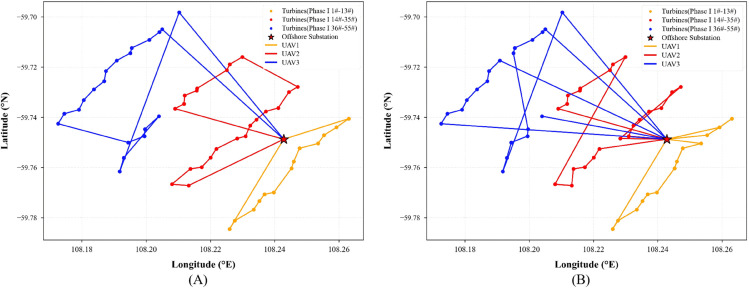
(A) Initial path planning result using the OPTION-A*-DQN algorithm. (**B**) Optimized path generated by the improved OPTION-A*-DQN algorithm.

Panels (A) of Figs 6, 7, and 8 present the initial path planning results for these three algorithms. It can be observed that OPTION-Dijkstra-DQN and OPTION-A*-DQN exhibit good task allocation performance but suffer from insufficient return-to-charge frequency planning. OPTION-SA-DQN, while achieving a certain level of coverage, shows apparent path fragmentation and redundancy, making it unsuitable for real-world engineering applications.

To address these issues, the reward function within the Markov Decision Process framework is redesigned with a focus on three aspects: (1) a low-battery penalty coefficient is introduced to encourage rational return-to-charge behavior; (2) a dynamic discount factor is applied to enhance the representation of long-term rewards; and (3) a path-smoothness reward term is added to improve trajectory coherence and navigational rationality. As shown in panels (B) of Figs 6, 7, and 8, the optimized results reveal significant improvements in path structure and return planning across three enhanced algorithms. Among them, OPTION-A*-DQN delivers the best performance in terms of path continuity and global optimality; OPTION-Dijkstra-DQN achieves adequate coverage but still requires improvements in return-to-charge logic; and although OPTION-SA-DQN resolves the coverage issue, it suffers from excessive path redundancy, with up to 19.2% of the trajectory being inefficient, limiting its overall effectiveness.

**Fig 9:**
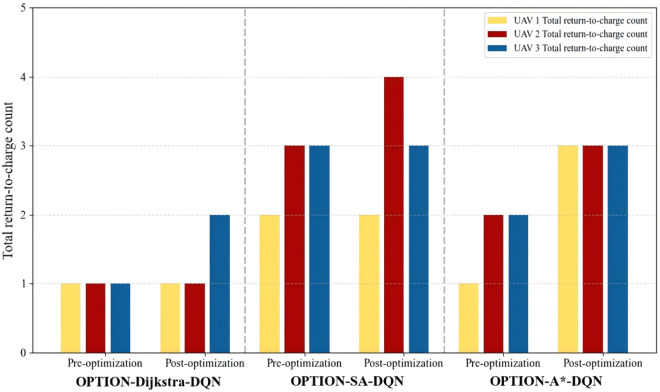
Bar chart comparing the return-to-charge frequency before and after optimization for different algorithms.

To further validate the impact of the proposed path optimization strategy on UAV return-to-charge behavior, this study statistically analyzes the number of return events before and after optimization across the three hybrid algorithms, with the results presented in the form of bar charts (see Fig 9). The results indicate that all algorithms exhibited varying degrees of increase in return frequency after the introduction of low-battery penalty and path-smoothness reward mechanisms. Specifically, return-to-charge frequency in the OPTION-A*-DQN model significantly increased from 5 to 9, demonstrating its superior performance in energy management strategies. Similarly, OPTION-Dijkstra-DQN and OPTION-SA-DQN increased from 3 to 4 and from 8 to 9 return events, respectively.

These findings suggest that the optimized path planning algorithms not only enhance inspection coverage but also better align with the practical operational requirements of offshore wind farms, which demand segmented inspection and intelligent return-to-charge behavior. Consequently, the system’s continuity and operational stability are substantially improved.

### Simulation testing phase

To systematically evaluate the impact of different heuristic algorithms on the path optimization performance of OPTION-DQN, this section constructs a simplified wind farm model within a small-scale simulation environment. A total of 30 wind turbine coordinates were randomly generated for controlled experiments. Preliminary results (see Table 2) indicate that the original OPTION-DQN suffers from two significant limitations: (1) a high redundant inspection rate of up to 20%, leading to inefficient path utilization and reduced system performance; and (2) frequent omission of turbine inspections, posing serious risks to task completeness and operational safety. These findings highlight inherent deficiencies in task allocation and path coordination mechanisms.

**Table 2:** Comparison of algorithm performance under random coordinate points.

Algorithm	Total Rewards	Path Distance (km)	Simulation Time	Repeat Access Points	Unreachable Points
OPTION-DQN	–1002.2	202.2	223.2	6	3
OPTION-SA-DQN	-1938.4	173.5	183.3	0	0
OPTION-Dijkstra-DQN	332.3	77.7	87.7	0	0
OPTION-A*-DQN	334.7	75.3	85.2	0	0

As shown in Table 2, OPTION-Dijkstra-DQN and OPTION-A*-DQN demonstrated the best performance in terms of convergence speed and path efficiency, with episode returns increasing by approximately 1.33 times, task completion rate increased by 10%, path distance reduced by over 61%, and simulation time decreased by around 60%, while maintaining strong training stability.

**Fig 10:**
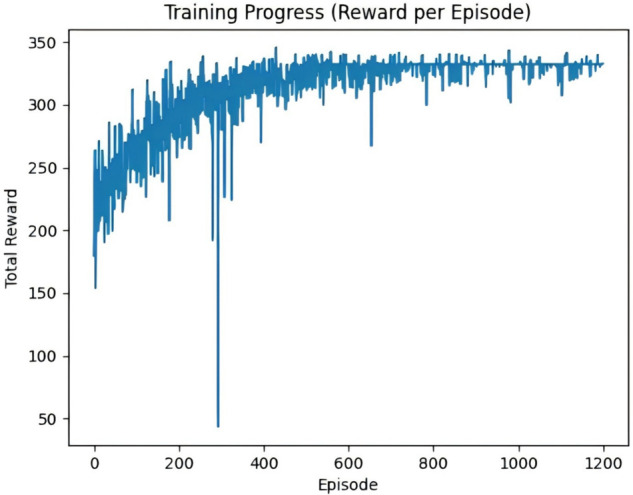
OPTION-Dijkstra-DQN algorithm cycle payoff plot.

**Fig 11:**
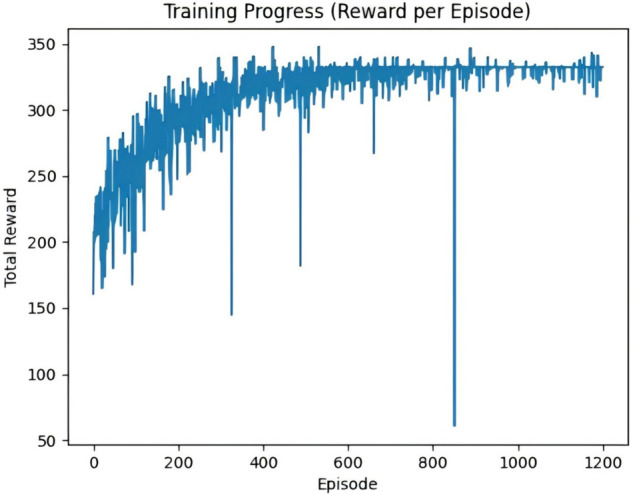
OPTION-A*-DQN algorithm cycle payoff plot.

Figs 10 and 11 illustrate the episodic return curves of the OPTION-Dijkstra-DQN and OPTION-A*-DQN algorithms under a simulated scenario with 30 randomly distributed wind turbine coordinates. Overall, both improved algorithms exhibit a steadily increasing trend in episodic returns, indicating that integrating heuristic path guidance mechanisms significantly enhances the performance and stability of path planning. Most return curves are closely clustered, reflecting consistent optimization effects across different training episodes.

However, a few curves demonstrate noticeable drops during specific iterations, primarily due to large penalty values imposed during UAV return-to-charge actions triggered by low battery levels. These penalties introduce temporary fluctuations in return values. Despite this, the overall trend confirms that the improved algorithms effectively boost task execution efficiency and enhance the robustness of path optimization performance.

### Engineering testing phase

To evaluate the applicability of the improved algorithms in real-world engineering scenarios, this study employed the actual coordinates of 55 wind turbines from the Phase I Yangjiang offshore wind farm as test data for path planning validation. Given that the original OPTION-DQN and OPTION-SA-DQN algorithms exhibited unstable convergence behavior during training and underperformed in small-scale experiments, only the OPTION-Dijkstra-DQN and OPTION-A*-DQN models were subjected to full-scale testing.

As shown in Table 3, compared to OPTION-Dijkstra-DQN, OPTION-A*-DQN achieves a 14.9% reduction in path distance and a 20% improvement in time efficiency, further confirming its engineering applicability in complex offshore environments.

**Table 3:** Comparison of algorithm performance under actual coordinate points.

Algorithm	Total Rewards	Unit Distance	Unit Time
OPTION-Dijkstra-DQN	877	0.47	10
OPTION-A*-DQN	3007	0.40	8

## Conclusions

This study presents an intelligent UAV path planning method for offshore wind farm inspections, integrating the OPTION-A*-DQN hybrid algorithm with an improved K-Means clustering algorithm and also establishing a four-dimensional constraint model. The proposed method demonstrates three key advancements: (1) a 10% improvement in task completion rate compared to baseline approaches, (2) a 14.9% reduction in path distance through optimized global-local navigation balance, and (3) a 20% decrease in simulation time enabled by efficient multi-UAV collaboration.

The proposed OPTION-A*-DQN algorithm significantly improves inspection efficiency, reduces path distance, and enhances task completion rates by optimizing UAV trajectories and multi-agent coordination in offshore wind farms. This provides wind farm operators with a robust decision-support solution, contributing substantially to the advancement of intelligent inspection systems while promoting operational safety and maintenance cost reduction. Future research will focus on further improving the algorithm’s adaptability by integrating transfer learning and meta-learning paradigms to enable efficient performance across diverse wind farm configurations and dynamic weather conditions.
